# Nanoparticles reduce monocytes within the lungs to improve outcomes after influenza virus infection in aged mice

**DOI:** 10.1172/jci.insight.156320

**Published:** 2022-08-08

**Authors:** William J. Kelley, Kathleen M. Wragg, Judy Chen, Tushar Murthy, Qichen Xu, Michael T. Boyne, Joseph R. Podojil, Adam Elhofy, Daniel R. Goldstein

**Affiliations:** 1 Department of Internal Medicine and; 2 Graduate Program in Immunology, University of Michigan, Ann Arbor, Michigan, USA.; 3 Research and Development, COUR Pharmaceuticals Development Company Inc., Northbrook, Illinois, USA.; 4 Department of Microbiology and Immunology, University of Michigan, Michigan, USA.

**Keywords:** Aging, Infectious disease, Influenza, Innate immunity

## Abstract

Older people exhibit dysregulated innate immunity to respiratory viral infections, including influenza and SARS-CoV-2, and show an increase in morbidity and mortality. Nanoparticles are a potential practical therapeutic that could reduce exaggerated innate immune responses within the lungs during viral infection. However, such therapeutics have not been examined for effectiveness during respiratory viral infection, particular in aged hosts. Here, we employed a lethal model of influenza viral infection in vulnerable aged mice to examine the ability of biodegradable, cargo-free nanoparticles, designated ONP-302, to resolve innate immune dysfunction and improve outcomes during infection. We administered ONP-302 via i.v. injection to aged mice at day 3 after infection, when the hyperinflammatory innate immune response was already established. During infection, we found that ONP-302 treatment reduced the numbers of inflammatory monocytes within the lungs and increased their number in both the liver and spleen, without impacting viral clearance. Importantly, cargo-free nanoparticles reduced lung damage, reduced histological lung inflammation, and improved gas exchange and, ultimately, the clinical outcomes in influenza-infected aged mice. In conclusion, ONP-302 improves outcomes in influenza-infected aged mice. Thus, our study provides information concerning a practical therapeutic, which, if translated clinically, could improve disease outcomes for vulnerable older patients suffering from respiratory viral infections.

## Graphical abstract

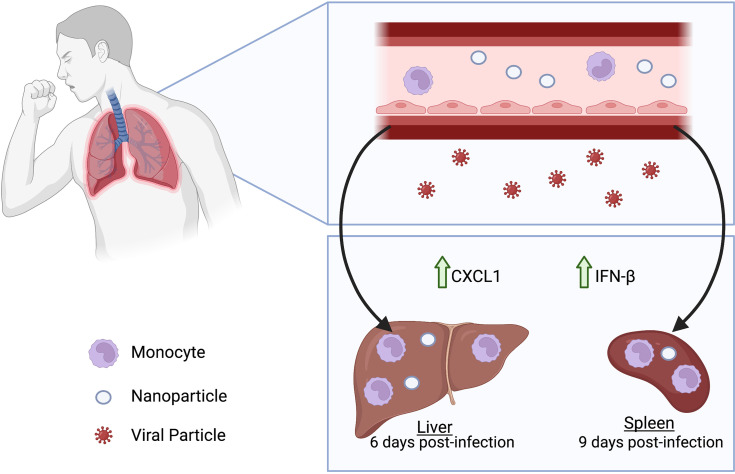

## Introduction

Older people are at a substantial increased risk of death from respiratory viral pathogens, including both the influenza virus and SARS-CoV-2 virus (1). This is due in part to aging’s complex effects on the adaptive immune system, including the reduction of naive T cell function, accumulation of memory T cells, impairment of follicular T cells, and reduction of antibody production (2–5). Aging also dysregulates the innate immune system, with evidence of an increased basal inflammatory responses (6, 7) coupled to defective innate immune activation upon stimulation (8–10). With influenza viral infection, studies in mice have shown that aging impairs the phagocytic function of alveolar macrophages (AMs) (11), key innate sentinel cells, and this leads to pathological accumulation of neutrophils within the lungs during infection (12). This exuberant innate immune response also occurs experimentally in murine models of coronavirus infection, as well as clinically, especially in older patients who succumb to COVID-19 (13).

Typically, the innate immune response is the first line of defense during viral infection such as infection from the influenza virus (14). As the host cells, including epithelial cells, are activated by the virus, these cells produce type I IFNs, inflammatory cytokines, and chemokines (14). These inflammatory cytokines and chemokines result in the recruitment of innate immune cells, including neutrophils, monocytes, and NK cells, to clear infected cells from the lungs. These innate immune cells rapidly transmigrate from the lung vasculature into the airways and remove dead and infected cells via efferocytosis; in the case of neutrophils, they also promote the release of cytotoxic materials, including myeloperoxidase (MPO) and neutrophil extracellular traps (NETs) (15–17). This process is part of the physiological innate immune response to infection and helps to clear the influenza virus from the host (18–20). However, aging dysregulates this innate immune response, leading to a massive influx of innate immune cells, such as neutrophils and monocytes, which leads to damaging immunopathology (7, 12). This results in a permeable lung vasculature, which impairs gas exchange, leading to morbidity and mortality, especially in older people (21, 22). Effective therapeutics are needed to reduce immune pathology and improve outcomes in older people infected with respiratory viruses.

Recent work in the field of nanoparticle-based drug delivery suggests that nanoparticle administration may ameliorate an excessive innate immune response by diverting inflammatory innate immune cells from the site of tissue injury to the liver and spleen (23). The administration of negatively charged, biodegradable, cargo-free nanoparticles, ONP-302, has been shown to improve disease outcomes in murine models of myocardial infarction, experimental autoimmune encephalomyelitis, West Nile virus–induced encephalitis, spinal cord injury, and metastatic breast cancer by diverting and/or “reprogramming” inflammatory innate immune cells away from the site of tissue damage (24–27). In every model of acute inflammation, ONP-302 prevented the infiltration of proinflammatory myeloid cells, including monocytes, macrophages, and neutrophils, into sites of pathology, leading to improved survival, tissue repair, and functional recovery (24–26). Importantly, ONP-302 did not cause broad immune suppression (24–27), which is important especially in older hosts who are already susceptible to infections. Although studies in mice have found that antibody-mediated depletion of neutrophils enhances survival during influenza infection with aging (12), this approach is not translatable to humans, as no monoclonal antibody for the depletion of human neutrophils exists. Hence, ONP-302 is a potentially attractive therapeutic option to improve outcomes to respiratory viral infection. Regrettably, no studies have demonstrated that ONP-302 are effective in aged hosts, especially in the context of acute respiratory viral infection. Demonstration that ONP-302 are effective in aged hosts is critical, as prior studies in mice have found that immune modulation may exhibit contrasting effects in young as compared with aged hosts, due to alterations in the immune system with aging (28).

In this study, we investigated whether the administration of ONP-302 nanoparticles would reduce the dysregulated hyperinflammatory innate immune response during influenza virus infection in aged mice. Given that humans present with symptoms at random times after infection, we chose to examine whether administration of nanoparticles are therapeutically effective when the pathophysiological inflammatory response to viral infection has already been established. Based on prior work (12), we hypothesized that the administration of nanoparticles beginning at 3 days postinfection (dpi), would reduce inflammation and lung injury in aged mice, resulting in reduced morbidity. Our study shows that ONP-302 administration reduced the accumulation of monocytes within the lungs and increased the numbers of monocytes within spleen and liver in aged mice during acute influenza infection. Additionally, ONP-302 treatment significantly improved the oxygen saturation levels and reduced morbidity of treated, infected aged mice compared with age-matched, infected controls. Collectively, our results show that the administration of ONP-302 nanoparticles improves outcomes in influenza-infected aged mice and reduces the number of innate monocytes within lungs. These results have major implications for the development of therapeutics for vulnerable hosts, particularly older people, during influenza infections and potentially during other respiratory viral infections, such as respiratory syncytial virus (RSV) and SARS-CoV-2.

## Results

### Nanoparticle fabrication and characterization.

**Figure 1:**
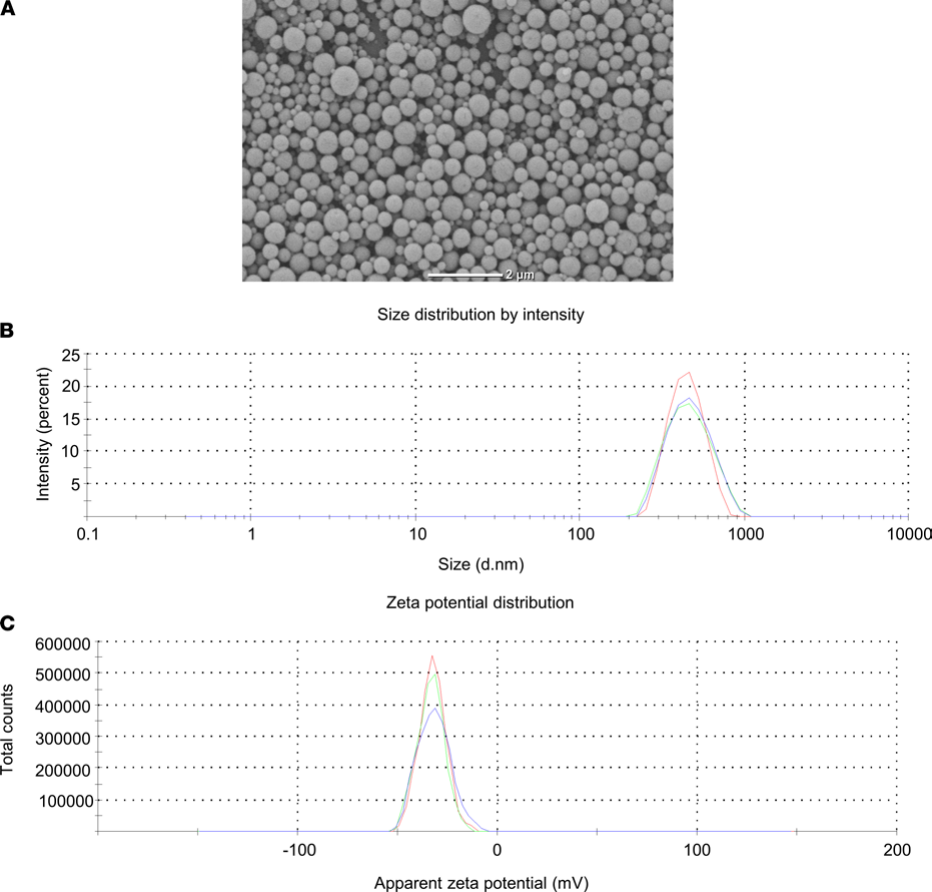
Physical characterization of ONP-302 by scanning electron microscopy. (**A**) ONP-302 nanoparticles were characterized by scanning electron microscopy. Shown is a representative image at 10,000× magnification depicting smooth and intact spherical particles. Scale bar: 2 μm. ONP-302 size and ζ potential were determined by dynamic light scattering (DLS). (**B** and **C**) Shown are DLS plots for size (nm) (**B**) and ζ potential (mV) (**C**) measurements from 3 readings.

ONP-302, cargo-free nanoparticles, were made from poly (lactic-co-glycolic) acid and manufactured using a double-emulsion technique as described in Methods. The ONP-302 manufacturing process was optimized for obtaining particles with an average diameter of 400–800 nm and a negative ζ potential of < –30 mV. ONP-302 physiochemical properties were characterized by dynamic light scattering (DLS) and electron microscopy (Figure 1). As shown in Table 1, ONP-302’s average size was 436.9 nm, with a ζ potential of –32.5 mV.

### ONP-302 administration reduces the accumulation of monocytes in the lung but increases the numbers of monocytes in the liver and spleen during IAV infection.

To investigate the impact of ONP-302 on innate immune cell accumulation in the lung during influenza infection with aging, aged (18–22 months of age) C57BL/6J mice were infected intranasally with 600 PFU H1N1 Influenza A VR-95 (IAV). Mice were treated with either 1 mg of ONP-302 or vehicle control via daily tail vein injections from 3 dpi to 5 dpi or 7 dpi. We chose 3 dpi, as this was when there was a 50-fold increase in myeloid cell infiltration within the lungs (Supplemental Figure 1; supplemental material available online with this article; https://doi.org/10.1172/jci.insight.156320DS1) (12). In age-infected mice that were treated with ONP-302 from 3 to 5 dpi, we harvested lungs, bronchiolar lavage fluid (BALF), liver and spleen at 6 dpi, and their cellular contents were analyzed via flow cytometry (for our flow cytometric gating strategy, see Supplemental Figure 2). We also included another cohort of aged-infected mice that were treated with ONP-302 from 3 to 7 dpi, and we collected the above tissues at 9 dpi. In either case, we included vehicle control–treated age-infected mice.

**Figure 2:**
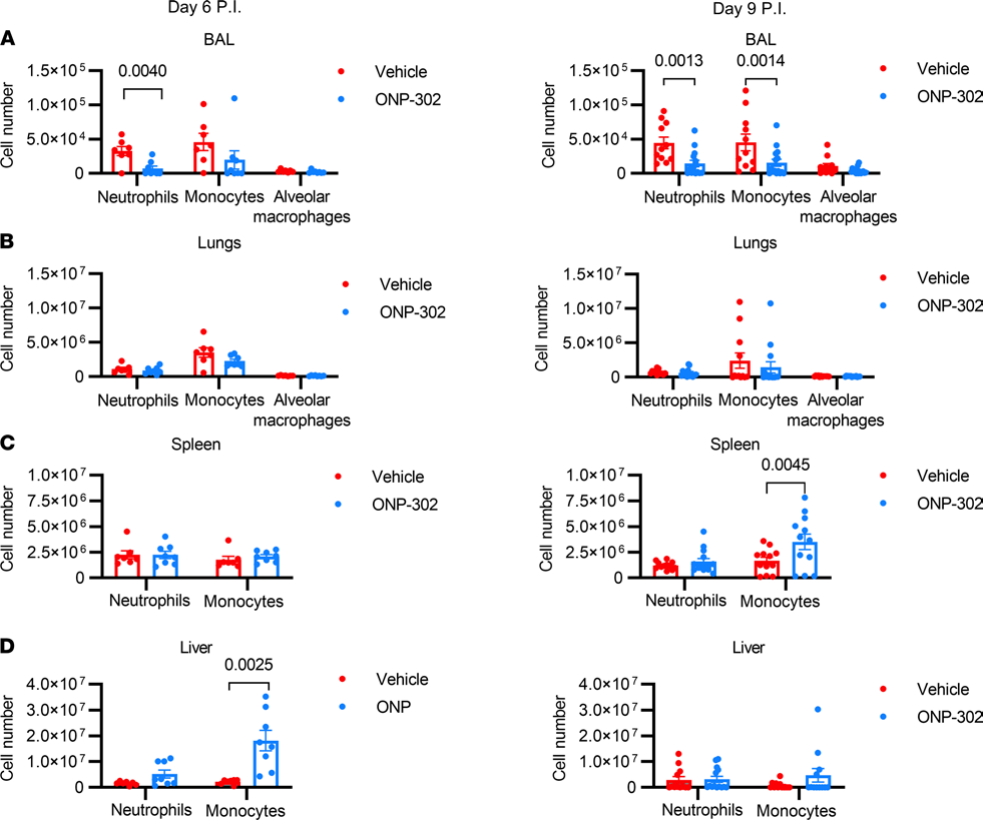
ONP-302 administration reduced myeloid cell accumulation in the lungs during influenza infection in aged mice. Aged female (18–20 months of age) C57BL/6J mice were infected with 600 PFU IAV then administered ONP-302 daily, or vehicle control, via i.v. tail vein injection from day 3 dpi to either day 5 dpi or day 7 dpi. In aged mice that received ONP-302 from 3 to 5 dpi, tissue was harvested on 6 dpi. For aged mice given ONP-302 from 3 to 7 dpi, tissue harvest was at 9 dpi. Total cell numbers are displayed in each graph as determined by flow cytometry. (**A**) BAL at 6 dpi or 9 dpi. (**B**) Lungs at 6 dpi or 9 dpi. (**C**) Spleen at 6 dpi or 9 dpi. (**D**) Liver at 6 dpi or 9 dpi. Data are expressed as mean ± SEM. Each data point represents a separate biological replicate. Data at 6 dpi represent 1 experiment with *n* = 8/experimental group. For 9 dpi, data represent samples pooled from 3 independent experiments with a total group size of *n* = 9/experimental group. Statistical significance was determined using Student’s *t* test. Significant *P* values are shown.

As shown in Figure 2, when we examined age-infected mice at 6 dpi, we found that there was a 3- to 4-fold reduction (*P* = 0.004) in the number of neutrophils, but not monocytes, within the BALF in aged mice that were treated with ONP-302 as compared with vehicle controls (Figure 2A). Notably, no significant changes in neutrophil, monocyte, or AM counts were detected in the lung digest at either 6 or 9 dpi (Figure 2B). This is because ONP-302 administration primarily affects infiltrating innate immune cells, the vast majority of which are captured in the BAL. The findings at 6 dpi were accompanied by an increased accumulation of monocytes within the liver (*P* = 0.0025), but not the spleen, of ONP-302–treated aged mice as compared with vehicle control, age-infected mice (Figure 2, C and D). We did not observe significant increases in absolute neutrophil numbers within the liver and spleen at this time point in comparing age-infected mice that received ONP-302 with those that received vehicle control (Figure 2, C and D).

When we examined age-infected mice at 9 dpi, we found that ONP-302 administration reduced the accumulation of both neutrophils (*P* = 0.0013) and monocytes (*P* = 0.0014) in the BALF by ~3-fold compared with vehicle controls. We found that particle administration resulted in a 2-fold increase in monocyte counts in the spleen (*P* = 0.0045) (Figure 2C) and a nonsignificant increase in monocyte counts in the liver (Figure 2D).

Collectively, when we examined mice at either 6 or 9 dpi, we found that even after the inflammatory response to infection was established, ONP-302 administration still reduced the number of neutrophils and monocytes within the BAL of aged, influenza-infected mice and increased the accumulation of monocytes both in the liver (at 6 dpi) and spleen (at 9 dpi).

### ONP-302 reduces the numbers of Ly6c^hi^ monocytes in the lung with increased accumulation of Ly6c^hi^ monocytes in the liver during IAV infection in aged mice.

**Figure 3:**
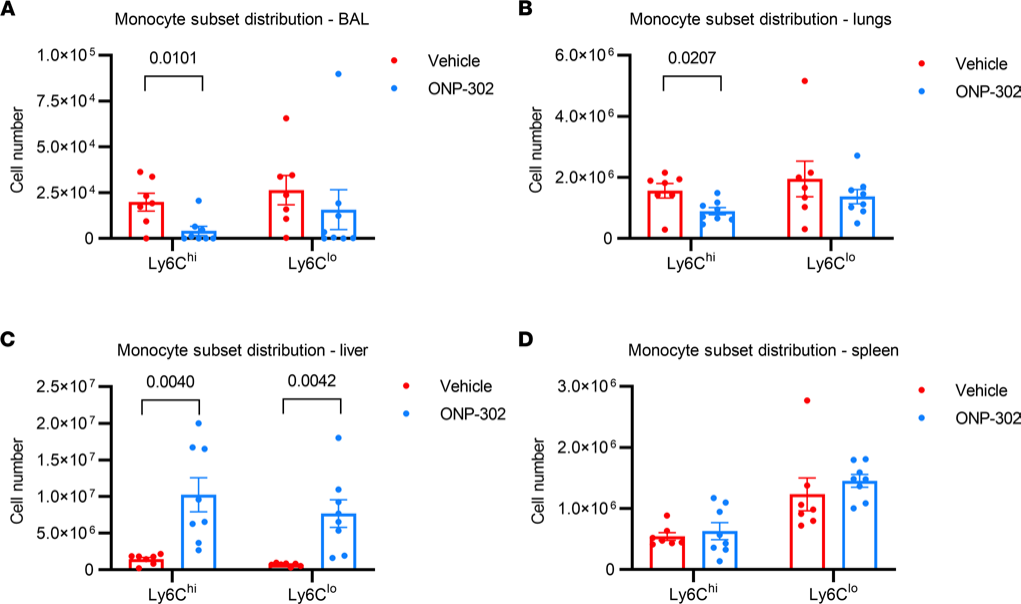
ONP-302 administration reduced Ly6C^hi^ monocyte numbers in the lung during IAV infection in aged mice. Aged female C57BL/6J mice were infected with 600 PFU IAV and were then administered ONP-302 daily, or vehicle control, via i.v. tail vein injection from 3 to 5 dpi. Tissue was harvested at 6 dpi. (**A**) Absolute number of Ly6C^hi^ and Ly6C^lo^ monocytes in the BAL. (**B**) Absolute number of Ly6C^hi^ and Ly6C^lo^ monocytes in the lung. (**C**) Absolute number of Ly6C^hi^ and Ly6C^lo^ monocytes in the liver. (**D**) Absolute number of Ly6C^hi^ and Ly6C^lo^ monocytes in the spleen. Data are expressed as mean ± SEM. Each data point represents a separate biological replicate — i.e., *n* = 8/group. Statistical significance was determined using Student’s *t* test. Significant *P* values are shown.

Monocytes can be divided into inflammatory (i.e., Ly6c^hi^ monocytes) and patrolling monocytes (i.e., Ly6c^lo^ monocytes) (29–31). We next examined if ONP-302 impacted the accumulation of inflammatory and patrolling monocytes in aged-influenza infected mice. As described above, aged mice were treated with 1 mg of ONP-302 or vehicle control via daily tail vein injections from 3 dpi to 5 dpi. Mice were then euthanized at 6 dpi We found that aged mice that were administered ONP-302 exhibited significant, 2- to 3-fold reduction of inflammatory, but not patrolling, monocytes in both the lung (*P* = 0.02) and BALF (*P* = 0.01) as compared with mice treated with vehicle control (Figure 3, A and B). At the same time point after infection, we noted that aged mice treated with ONP-302 exhibited a significant 2- to 3-fold increase in the numbers of both inflammatory and patrolling monocytes in the liver (*P* = 0.004) but not the spleen as compared with infected aged mice administered vehicle control (Figure 3, C and D). These data indicate that ONP-302 impairs the accumulation of inflammatory monocytes into the lung of age-influenza infected mice and increases the numbers of monocytes in the liver.

Since monocytes can initiate adaptive immune responses, specifically CD4^+^ T cells (32, 33), we next examined if the administration of ONP-302 impacted the number of T cells and B cells in the BAL after influenza infection. After administering ONP-302, or vehicle control, from 3 dpi to 5 dpi, at 6 dpi, we did not observe any alterations in the absolute numbers of T cells, B cells, CD4^+^ T cells, and CD8^+^ T cells within the BAL between the ONP-302–treated mice and vehicles controls (Supplemental Figure 3). These results indicate that, whereas ONP-302 impacts the numbers of monocytes and neutrophils in the BALF at 6 dpi, it does not impact the numbers of T and B cells.

### ONP-302 administration improves oxygen saturation and clinical score in aged, influenza-infected mice.

Next, we sought to evaluate the therapeutic impact of particle administration on influenza infection in aged mice. Toward this end, we monitored infected mice daily for oxygen saturation levels and clinical disease progression (12). Specifically, we used pulse oximetry (Figure 3A) and clinical scoring (Figure 3B) to evaluate disease progression in both particle-treated and vehicle-treated mice. Note that, when we infused ONP-302 into noninfected aged mice, this did not impact oxygen saturation levels (Supplemental Figure 4A). Also, in aged mice that were not infected but received ONP-302, we failed to observe an increase in monocytes and neutrophils within the BAL as compared with vehicle-treated mice (Supplemental Figure 4B).

**Figure 4:**
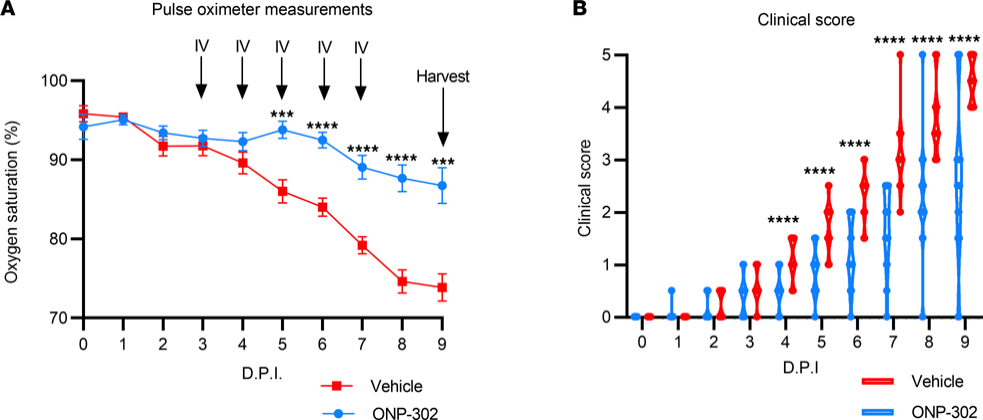
ONP-302 administration improves oxygen saturation and clinical score in aged, influenza-infected mice. (**A**) Oxygen saturation levels in ONP-302–treated and vehicle-treated aged mice during influenza infection, as measured by pulse oximeter (MouseSTAT Jr.). (**B**) Clinical scoring of ONP-302–treated and vehicle-treated aged mice after influenza infection. Aged female C57BL/6J mice were infected with 600 PFU IAV, followed by 5 daily tail vein injections of vehicle or ONP-302 beginning at 3 dpi. Mice were euthanized at 9 dpi. *n* ≥ 22/group. Data are expressed as mean ± SEM. Data represent mice pooled from 3 independent experiments. Statistical significance was determined using Student’s *t* test with Bonferroni correction. ***P < 0.005; ****P < 0.0005.

As shown in Figure 4A, both particle-treated and vehicle-treated mice exhibited decreased oxygen saturation over the initial course of the infection. However, whereas the oxygen saturation levels of vehicle-treated aged mice decreased from an oxygen saturation level of approximately 95% at 0 dpi to approximately 74% at 9 dpi, particle-treated aged mice were able to maintain their oxygen saturation levels > 87% during the entire course of the infection (Figure 4A). In agreement with the pulse oximetry results, particle-treated aged mice exhibited a significantly lower clinical disease score beginning at 4 dpi and continuing throughout the remainder of the infection (Figure 4B). At 9 dpi, particle-treated aged mice exhibited an average clinical score of approximately 2.5, compared with an average clinical score of approximately 4.5 (for details of the scoring system, see Methods) for vehicle-treated mice. Taken together, these results suggest that particle administration improves gas exchange and reduces morbidity in influenza-infected aged mice.

### ONP-302 administration reduced neutrophil activation, lung damage, and inflammatory cytokine levels in the lungs during influenza infection with aging.

In order to further probe the effects of ONP-302 administration on lung damage and inflammation, as well as viral replication during infection with aging, we measured the activation of neutrophils in addition to measuring the concentration of a range of proteins and cytokines in the BALF via ELISAs, as well as the viral loads via a viral plaque.

**Figure 5:**
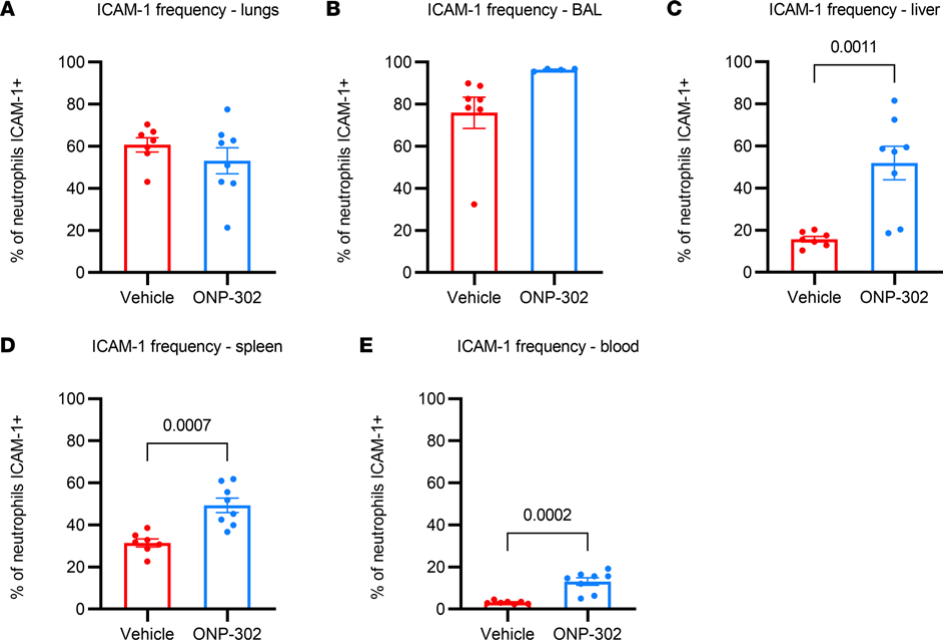
ONP-302 increases the number of ICAM^+^ neutrophils in the liver, spleen, and blood during IAV infection in aged mice. Aged female C57BL/6J mice were infected with 600 PFU IAV and were then administered ONP-302 daily, or vehicle control, via i.v. tail vein injection from 3 to 5 dpi. Tissue was harvested at 6 dpi. (**A**) ICAM^+^ neutrophils in the lung. (**B**) ICAM^+^ neutrophils in the BALF. (**C**) ICAM^+^ neutrophils in the liver. (**D**) ICAM^+^ neutrophils in the spleen. (**E**) ICAM^+^ neutrophils in the blood. Data are expressed as mean ± SEM. Each data point represents a separate biological replicate — i.e., *n* = 8 mice/group. Statistical significance was determined using Student’s *t* test. Significant *P* values are shown.

We first assessed the frequency of ICAM-1 neutrophils, as ICAM-1 correlates with enhanced effector function of neutrophils (34), in aged infected mice that were administered ONP-302, or vehicle control, from 3 dpi to 5 dpi. We then assessed the proportion of ICAM-1^+^ neutrophils in the BAL, lung, liver spleen, and blood (Supplemental Figure 2). We found that the ONP-302–treated aged mice exhibited a 2- to 3-fold increase in the frequency of ICAM-1^+^ neutrophils in the liver (*P* = 0.001), spleen (*P* = 0.0007), and blood (*P* = 0.002) but not the lung or BAL as compared with vehicle control aged mice (Figure 5 and Supplemental Figure 5). These data indicate that ONP-302 induces the accumulation of activated neutrophils within the blood, spleen, and liver at 6 dpi

**Figure 6:**
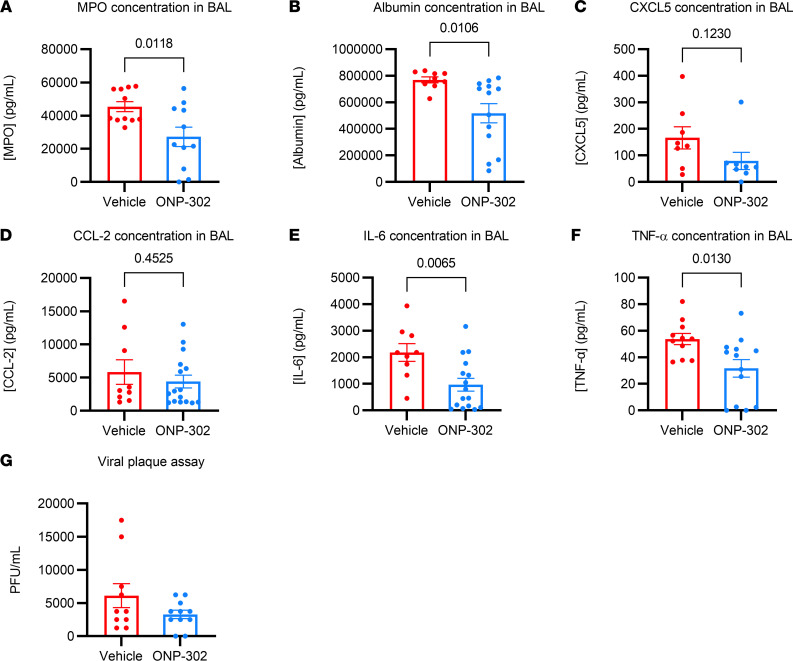
ONP-302 administration reduces neutrophil activation, lung damage, and the secretion of inflammatory cytokines into the BAL during influenza infect with aging. Aged female C57BL/6J mice were infected with 600 PFU IAV, followed by 5 daily tail vein injections of vehicle or ONP-302 beginning at 3 dpi. Mice were euthanized at 9 dpi. (**A**–**F**) Myeloperoxidase (MPO), albumin, CXCL5, CCL-2, IL-6, and TNF-α concentrations in the BAL obtained at 9 dpi measured by ELISA. (**G**) Influenza viral loads in the BAL as measured by viral plaque assay. Each data point represents a biological replicate. Data are expressed as mean ± SEM. Data represent samples pooled from 3 independent experiments. Statistical significance was determined using Student’s *t* test. Significant *P* values are shown.

We next examined the levels of MPO in aged mice that were administered ONP-302 from 3 dpi to 7 dpi and analyzed lung samples at 9 dpi. MPO is crucial to the bactericidal and phagocytic functions of neutrophils, and excessive MPO release has been shown to cause oxidative stress, DNA damage, and cell death in alveolar and bronchial epithelial cells (35, 36). ONP-302–treated aged mice exhibited an approximately 2-fold reduction in MPO concentration compared with vehicle-treated aged mice (*P* = 0.0118) at 9 dpi (Figure 6A). In conjunction, ONP-302–treated aged mice exhibited a significantly reduced concentration of albumin, a marker of permeable lung vasculature, in the BALF (~30% reduction, *P* = 0.0106) compared with vehicle-treated aged mice (Figure 6B). Thus, taken with the significant decrease in MPO concentration, the reduction in albumin concentration for particle-treated mice suggests significant attenuation of lung injury during infection.

We did not find a significant reduction in the chemokine CCL-2, which recruits monocytes, or the chemokine CXCL5, which recruits neutrophils (37, 38) (Figures 6, C and D). This suggests that ONP-302 administration does not impede with the active recruitment of myeloid cells into the lung via chemokine signaling pathways. Rather, the particles may cause the reduction of inflammatory monocytes and neutrophils in the lungs via a different mechanism.

Additionally, we measured a significant decrease in the IL-6 concentration in the BAL, an inflammatory cytokine that correlates with disease outcomes in acute respiratory infections, including both influenza and COVID-19, in humans (39–42). We found that ONP-302–treated aged mice exhibited an approximate 2-fold reduction in IL-6 compared with age-matched infected mice treated with vehicle control (*P* = 0.0065) (Figure 6E). Similarly, we found that ONP-302 treatment led to a significant 2-fold reduction in another inflammatory cytokine, TNF-α, in the BALF of aged, influenza-infected mice (*P* = 0.0130) (Figure 6F) as compared with controls. These findings suggest that, by reducing inflammatory monocytes within the lungs, we dampened the inflammatory response to influenza infection in aged mice administered ONP-302.

Finally, we used a viral plaque assay to measure the viral loads in the BAL of both ONP-302–treated and vehicle-treated mice at 9 dpi. Notably, we found no difference in the viral loads between ONP-302–treated and vehicle-treated aged mice (Figure 6G). This finding indicates that ONP-302 treatment has no direct effect on viral replication within the BAL. Our results suggest that ONP-302 reduces the inflammatory innate immune response to influenza infection independently of viral clearance in aged mice during infection.

### ONP-302 administration reduces cellular infiltrates and lung damage.

**Figure 7:**
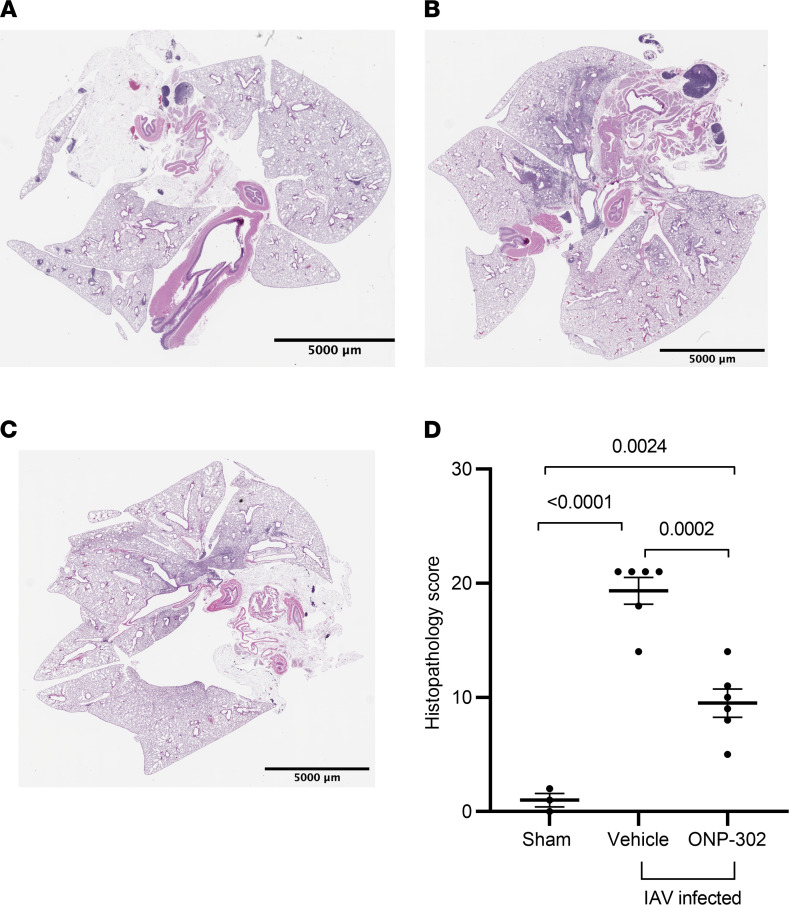
ONP-302 administration reduces histological evidence of lung inflammation during influenza infection in aged mice. Aged female C57BL/6J mice were infected with 600 PFU IAV, followed by 5 daily tail vein injections of vehicle or ONP-302 beginning at 3 dpi. Mice were euthanized at 9 dpi. (**A**) Sham (PBS) infected control (9 dpi). (**B**) Influenza-infected, vehicle-treated aged mouse (9 dpi). (**C**) Influenza-infected, ONP-302–treated aged mouse (9 dpi). Histological samples are representative of 2 independent experiments with at least 3 mice/group/experiment. (**D**) Quantification of histology for each experimental group read blindly. Statistical significance was determined using 1-way ANOVA with Tukey’s multiple comparison. Each point indicates a biological replicate. Scale bars: 5000 μm.

In order to visualize the degree of lung damage in vehicle-treated and ONP-302–treated aged mice, we performed histopathological analysis on the lungs of particle- and vehicle-treated groups at 9 dpi via H&E staining (Figure 7). As shown in Figure 7A, sham-infected aged mice exhibited healthy lungs with little damage and few cellular infiltrates. Influenza-infected, vehicle-treated aged mice, on the other hand, exhibited extensive lung damage and cellular infiltration, as evidenced by the H&E staining (Figure 7B). The lungs of ONP-302–treated aged mice, however, exhibited reduced numbers of cellular infiltrates compared with the vehicle-treated, infected aged mice (Figure 7C). Lung tissue of aged mice treated with ONP-302 exhibited decreased numbers of immune cells and RBCs within the alveoli and the interstitial space between the alveoli when compared with vehicle treatment. ONP-302 treatment decreased epithelial hyperplasia and the degree to which tissue histological structure was altered. When scored for lung damage, ONP-302–treated, infected aged mice exhibited lower scores as compared with vehicle-treated infected aged mice (*P* = 0.0001) (Figure 7D). These data indicate that ONP-302 reduce histological evidence of inflammation during influenza infection with aging.

### ONP-302 administration increases myeloid cell recruitment by tissue-resident macrophages in the spleen.

**Figure 8:**
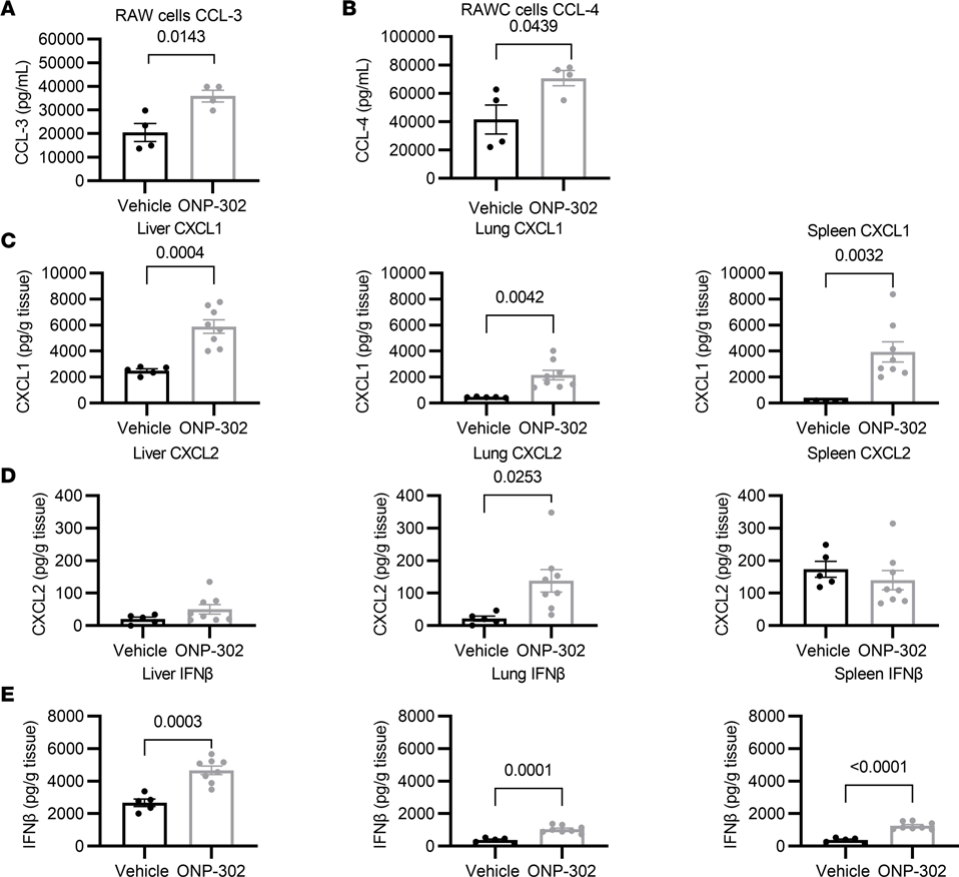
ONP-302 administration increases the production of myeloid cell chemoattractants in the spleen. (**A**) CCL-3 production by RAW cells exposed to ONP-302. (**B**) CCL-4 production by RAW cells exposed to ONP-302. (**C–E**) Aged noninfected mice were administered ONP-302, or vehicle control, for 5 days; 2 days after the fifth administration, the liver, lungs, and spleen were obtained, and CXCL1 (**C**), CXCL2 (**D**), and IFN-β (**E**) were measured via ELISA. Concentration of each factor was normalized to weight of tissue. Each data point represents a biological replicate. For **C** and **D**, *n* = 8 for ONP-302 group and *n* = 5 for vehicle control group. Data are expressed as mean ± SEM. Statistical significance was determined using Student’s *t* test. Significant *P* values are shown.

To investigate further the mechanisms by which ONP-302 administration reduces the number of monocytes in different peripheral tissues, we measured the production of chemoattractants by RAW 264.7 cells — a macrophage cell line — exposed to ONP-302, via ELISA. As shown in Figure 8, A and B, ONP-302 exposure significantly increased the production of CCL-3 (also known as MIP1-α) and CCL-4 (also known as MIP1-β) by RAW cells. Both CCL-3 and CCL-4 are well-known myeloid cell chemoattractants, suggesting that ONP-302 treatment may prime tissue-resident macrophages to increase the recruitment of myeloid cells (43–46). In order to probe this effect in vivo using our therapeutic regimen, we injected aged female C57BL/6J mice with either vehicle control or ONP-302 for 5 consecutive days. Two days after the last particle administration, we harvested and homogenized the spleen, lung, and liver and measured CXCL1, CXCL2, and IFN-β in the homogenate via ELISA. CXCL1 and CXCL2 are both involved in neutrophil and monocyte recruitment during infections and inflammation (12, 47–50). Additionally, IFN-β promotes the recruitment of myeloid cells to the spleen, limits myeloid cell–mediated damage in a murine model of cytomegalovirus, and resolves inflammation to play a protective role during influenza infection (51–54). As shown in Figure 8C, ONP-302 administration, as compared with vehicle control, increased the production by an approximate 2- to 5-fold increase of CXCL1 in the liver, lung, and spleen. ONP-302 also induced a 2- to 4-fold induction of IFN-β by the liver, lung, and spleen (Figure 8E). However, the induction of CXCL2 was only significantly elevated by the lung and not in the liver or spleen (Figure 8D). Please note that, for levels of CXCL1 and IFN-β, ONP-302 administration induced higher concentrations per gram of tissue from the liver as compared with the lung and spleen (compare ONP-302–induced levels in Figure 8, C and E, across the different organs). Thus, the administration of ONP-302 induces the production of CXCL1 and IFN-β, by peripheral organs, which may enhance the accumulation of monocytes in the liver and spleen during influenza viral infection.

## Discussion

Here, we employed negatively charged, biodegradable, cargo-free immunomodulatory nanoparticles — ONP-302 — as a potential therapeutic for influenza infection with aging. We hypothesized that nanoparticle administration would reduce the exaggerated inflammatory innate immune response to influenza infection in aged mice and, consequently, provide protection from the deleterious effects of neutrophil and monocyte infiltration during infection. To test our hypothesis, we administered the nanoparticles when myeloid cell accumulation into the lungs of aged hosts was established. This is important clinically, as humans are typically admitted to hospitals with acute respiratory viral infections after the initiation of the inflammatory response within the lungs. Importantly, humans > 65 years of age exhibit a substantial increase in mortality to acute respiratory viral infections, including both influenza infection and SARS-CoV-2 infection (13, 55, 56).

Our study found that daily particle administration from 3 dpi to either 5 dpi or 7 dpi reduced the accumulation of monocytes within the lungs and increased the numbers of monocytes in the liver and spleen (Figure 2). Importantly, nanoparticle administration also protected aged mice from infection-induced oxygen desaturation (Figure 4A) and improved clinical outcome during infection (Figure 4B). Furthermore, we found that ONP-302 reduced the levels of MPO, albumin, IL-6, and TNF-α in the lungs of aged, influenza-infected mice (Figure 6). Histopathological analysis confirmed that nanoparticle treatment reduced cellular infiltrates and lung damage in influenza-infected, aged mice (Figure 7). Our results demonstrate that cargo-free nanoparticles reduce morbidity in influenza-infected, aged mice.

We found that culturing ONP-302 with RAW macrophages led to the secretion of myeloid attracting chemokines (Figure 8, A and B). Furthermore, when we administered ONP-302 to aged, noninfected mice this led to the secretion of CXCL1 and IFN-β, which can enhance chemoattraction of myeloid cells (12, 47–54) (Figure 8, C–E). Our study suggests that macrophages in the liver and spleen are induced to secrete myeloid-attracting chemokines during particle treatment. Overall, our study suggests that, during influenza infection in aged mice, cargo-free nanoparticles reduce the accumulation of monocytes within lungs and increase the numbers of myeloid cells within the spleen and liver, possibly by increasing the release of chemoattracting signals by macrophages, independently of viral clearance.

Our study provides evidence that ONP-302 nanoparticles not only are effective in diverting inflammatory responses in young mice in alternative experimental scenarios outside of respiratory viral infection (24–27, 57–59) but are effective for treating acute respiratory viral infections in vulnerable aged mice. This therapeutic strategy is highly attractive due to its simplicity and to the manifold redundancies in immune signaling pathways. Blank, cargo-free nanoparticles are relatively simple to produce in large quantities, as they do not require complex biochemical modification or drug loading, which may ultimately result in a practical therapeutic platform. Furthermore, strategies focused on blocking a particular immune signaling pathway may be ineffective due to the large degree of redundancy within the immune system: if one innate immune pathway is blocked, other pathways compensate. Additionally, immunosuppressive therapies that target multiple pathways may compromise a patient’s immune system, preventing the patient from fighting the original infection or subsequent secondary bacterial infections (60–63). This is particularly relevant in older people who are at increased risk of secondary bacterial infections, especially in long-term care facilities (64).

Although our study indicates that ONP-302 reduces the numbers of myeloid cells in the lungs and increases the numbers of monocytes in the liver and spleen during influenza infection by potentially inducing the secretion of myeloid chemokines by the spleen and liver, there are likely multiple complex mechanisms in play. For example, previous work suggests that nanoparticles may interfere with myeloid cell extravasation, and, thus, lung infiltration, via multiple types of interactions. Physical collisions between particles and leukocytes in the bloodstream interfere with leukocyte adhesion to the vascular wall (57). A recent experimental study in young mice has also shown that nanoparticles may downregulate TLR activation on inflammatory cells, inhibiting the inflammatory response of innate immune cells and improve outcomes to endotoxemia (24). Although our study did not find that ONP-302–treated aged mice exhibited alterations in the number of adaptive immune cells within the BAL at 6 dpi, and that there was no difference in viral load at 9 dpi, it is possible that ONP-302 impacts adaptive immune responses at later time points after infection; this will require future investigation. Also, a prior study, in young mice, indicates that these nanoparticles activate the macrophage receptor with collagenous structure (MARCO) receptor on myeloid cells and induces apoptosis as the cells traffic to the spleen (26). Whether this is true in aged mice during acute respiratory viral infections should be investigated in the future. Although our study is compatible with the notion that ONP-302 diverts inflammatory monocytes away from the sites of inflammation to the spleen and liver, our study does not provide definitive proof of this. Such proof could require dynamic in vivo imaging in which one could simultaneously track the nanoparticles and myeloid cells during active respiratory viral infection in aged mice. This might require aging of specific transgenic mice in which myeloid cells express florescent proteins (65), and this might require development of approaches in which the lungs, spleen, and liver can be isolated for imaging while keep vulnerable aged mice alive during infection. Such complex approaches will require future investigation to decipher the complex in vivo effects of ONP-302 on myeloid cells during active infection.

Our preclinical study provides fundamental information that ONP-302 offers therapeutic advantages to vulnerable aged hosts during influenza viral infection. We employed the dosing regimen of ONP-302 based upon prior studies and experiences (26). Clearly, translation to humans will require future studies to show that ONP-302 are safe to administer prior to infection, and it will require optimization studies to identify the most effective dose to enhance outcomes during acute respiratory viral infections in humans. Although our study has indicated that ONP-302 nanoparticles improve clinical outcomes in vulnerable aged mice during influenza infection, other particles — either inorganic metal nanoparticles or organic particles such as liposomes and micelles — may also be effective (66, 67). Metal nanoparticles and organic particles have been typically employed to carry antiviral medications to improve outcomes to infections (67). Future studies will be required to determine the optimum dosing regimen of ONP-302 in humans and to evaluate other particle approaches to improve outcomes to respiratory viral infections with aging.

Overall, our study demonstrates that cargo-free nanoparticles improve outcomes of influenza viral infection in aged mice. These nanoparticles may prove to be a potential valuable therapeutic for acute respiratory distress syndrome induced by influenza, SARS-CoV-2, and potentially other respiratory viruses. Our study provides preclinical evidence of the efficacy of ONP-302 in aged mice, which should be translated clinically.

## Methods

### Particle preparation and characterization.

ONP-302 nanoparticles were manufactured by COUR Pharmaceuticals Development Company Inc. Particles were made from polylactic-co-glycolic acid (PLGA) (Lactel, Durect Corporation) using a double-emulsion technique. Briefly, a water-in-oil emulsion containing a proprietary mixture of PLGA and surfactants was prepared. Please note that 1 mg of ONP-302 refers to the PLGA content of the particles, which contains roughly 1 × 10^9^ particles. Solvents were removed by evaporation yielding negatively charged nanoparticles, which were then washed, filtered, and concentrated by tangential flow filtration. ONP-302 particles were then formulated with buffering agents and cryoprotectants and were lyophilized. ONP-302 physiochemical properties were characterized by DLS and scanning electron microscopy.

### Animals.

Aged (18–22 months) female C57BL/6J mice were purchased from the Jackson Laboratory and allowed to acclimate in our animal facility for 1 week prior to infection. One day prior to infection, mice were transported to a BSL-2 animal facility at the University of Michigan.

### Influenza infection.

H1N1 IAV was purchased from ATCC, aliquoted, and stored at –80°C until use. Viral titers were confirmed using viral plaque assays. Mice were anesthetized using isoflurane and inoculated intranasally with 600 PFU IAV in 40 μL of inoculum. After infection, mice were monitored daily for clinical score and oxygen saturation (MouseSTAT Jr., Kent Scientific). Clinical scores were determined as described previously. Briefly, a score of 0 indicated a normal, healthy mouse, 1 indicated slightly ruffled fur, 2 indicated ruffled fur, 3 indicated ruffled fur and inactive behavior, 4 indicated hunched back and moribund, and 5 indicated dead. The researcher who reported the clinical scores was not blinded to the experimental groups.

### Particle administration.

Particles were diluted to 5 mg/mL in sterile saline prior to injection. Beginning at 3 dpi, mice were administered 200 μL of either particle solution (1 mg/mouse) or vehicle control (i.e., saline at the same dilution factor as for the particles) via tail vein injection. Injections were continued daily for a total of 3 or 5 days (i.e., 3–5 or 3–7 dpi). This administration dose and route was based upon prior murine studies that employed ONP-302 (27).

### Euthanasia and sample collection.

At specific times after infection (3 dpi, 7 dpi, or 9 dpi, as noted), mice were euthanized via CO_2_ overdose. Mice were then dissected, and blood samples were collected into 0.1 mL heparin via cardiac puncture using a 23G needle. The trachea was exposed, a catheter was inserted into the trachea, and the lungs were lavaged twice with 1 mL of PBS without Ca^2+^ and Mg^2+^ as described previously to collect BALF (12). Finally, the lungs, liver, and spleen were collected for flow cytometric analyses.

### Preparation of single-cell suspensions for flow cytometry.

Mouse lungs and livers were digested into a single-cell suspension as previously described. Briefly, lungs and livers were injected with 3 mL of 1 mg/mL Collagenase D (Roche) with 100 U/mL DNase I (Roche). Lungs and livers were then minced with a razor blade and incubated at 37°C for 1 hour. Lungs and livers were then passed through 70 μm cell strainers (VWR International, catalog 76327-100) to obtain a single-cell suspension. Cells were then centrifuged at 500*g* for 5 minutes at 4°C and resuspended in 3 mL of 1× RBC lysis buffer (eBioscience) for 3 minutes. Lysis was quenched with an equivalent volume of FACS buffer (PBS w/ 2% FBS and 1 mM EDTA). Cells were then centrifuged at 500*g* for 5 minutes at 4ºC and resuspended in 20 μL of 1:40 TruStain FcX anti–mouse CD16/32 antibody (BioLegend, catalog 101320/clone 93) for 30 minutes on ice. Cells were recentrifuged at 500 g for 5 minutes at 4°C and stained with fluorescent antibodies for CD45 (BioLegend, 103114), CD11b (BioLegend, 101212), Ly6G (BioLegend, 127639), Ly6C (BioLegend, 128022), Siglec F (BD Biosciences, 747316), and ICAM-1 (BioLegend, 116141) on ice in the dark for 30 minutes. Alternatively, for enumeration of adaptive immune cells, lung and BAL samples were stained with fluorescent antibodies for CD45 (BioLegend, 103108), CD19 (BioLegend, 115537), CD3 (BioLegend #100236), CD4 (BioLegend, 100422), and CD8 (Invitrogen, 47-0081-82). Cells were centrifuged again at 500 g for 5 minutes at 4°C and resuspended in 1 mL of 4% paraformaldehyde (PFA) for 30 minutes at room temperature for fixation. Finally, cells were centrifuged at 500 g for 5 minutes at 4°C and resuspended in 500 μL of FACS buffer and stored at 4°C until analysis.

Mouse spleens were passed through 70 μm cell strainers by mechanical dissociation prior to RBC lysis, FC blocking, staining, and fixation steps as described above. Peripheral blood samples underwent RBC lysis followed by the FC blocking, staining, and fixation steps as described above. BAL samples were centrifuged at 500 g for 10 minutes at 4°C, the supernatants were aliquoted and stored at –80°C for ELISAs and viral plaque assays, and the cells underwent the FC blocking, staining, and fixation steps described above.

To determine total cell counts, a defined volume of antibody-stained single-cell suspension was collected by the flow cytometer. Recorded counts were adjusted based on tissue weight to reflect the entire tissue count or based on the total volume of fluid recovered for BAL samples.

### Flow cytometric analysis.

All samples were analyzed using a Bio-Rad ZE5 Cell Analyzer, and data were analyzed via FlowJo software. A representative flow cytometric gating strategy is shown in Supplemental Figure 2. Fluorescence minus one controls were used in all experiments for gating purposes. After gating for CD45^+^ singlets, neutrophils were identified as CD11b^+^Ly6G^+^, monocytes were identified as Ly6G^–^CD11b^+^SiglecF^–^Ly6C^+^, and AMs were identified as Ly6G^–^SiglecF^+^. B cells were identified as CD45^+^CD3^–^/CD19^+^, total T cells as CD45^+^ CD3^+^CD19^–^, CD4^+^ T cells as CD45^+^CD19^–^CD3^+^CD4^+^CD8^–^, and CD8^+^ T cells as CD45^+^CD19^–^CD3^+^CD4^–^CD8^+^. Samples with < 50 events recorded in the terminal population were excluded from further phenotypic analysis.

### Histopathology.

At 9 dpi, mice were euthanized via CO_2_ overdose. Mice were then perfused with ~10 mL of cold PBS. Then, the trachea was exposed, and a catheter was inserted as described above. Lungs were then inflated with ~1 mL of 10% buffered formalin, the trachea was tied off using a suture knot, and lungs were submerged in 3 mL of 10% buffered formalin for fixation. Samples were prepared for H&E staining and histopathological analysis at the In Vivo Animal Core at the University of Michigan. H&E-stained samples were examined microscopically to evaluate the degree of cellular infiltrate and lung damage qualitatively and quantitatively. Sections were scored by investigators blinded to the identity of the groups, for lung damage utilizing a graded scoring system (scores from 0 [no damage] to 3 [severe damage]) for 7 different histological features, which were summed to generate a final score. These histological features included the location and quantity of immune cells, location and quantity of RBCs (outside of the vasculature), epithelial hyperplasia, and the percentage of the lung tissue, as a whole, that showed signs of altered histological structure and tissue disruption.

### ELISAs.

Mouse MPO (catalog DY3667), CXCL1 (catalog DY453), and CXCL2 (catalog MM200) ELISA kits were purchased from R&D Systems. Mouse TNF-α (catalog BMS607-3), CCL2 (catalog 88-7391-88), CXCL5 (catalog EMCXCL5), and IL-6 (catalog KMC0061) ELISA kits were purchased from Invitrogen. The mouse albumin ELISA kit was purchased from Bethyl Laboratories (catalog E99-134). All ELISAs were stored and used per the manufacturer’s protocols. BAL samples were stored at –80°C prior to use in ELISAs, and samples were diluted in PBS to an appropriate concentration for each assay.

Spleen, lung, and liver lysates were prepared from cryopreserved tissues for assessment of analytes in tissue. Briefly, tissues were minced in T-PER tissue protein extraction reagent (Thermo Fisher Scientific, 78510) containing 1% protease inhibitor cocktail (MilliporeSigma, P8340) and 1% phosphatase inhibitor cocktail 2 (MilliporeSigma, P5726) prior to homogenization with an Omni Tissue Homogenizer TH (Omni International). The resulting homogenates were incubated on ice for 30 minutes and were then centrifuged at 12,000*g* for 10 minutes at 4°C. The supernatants were collected and stored at –80°C prior to use. Analyte content was normalized to the weight of each tissue.

### Viral plaque assay.

Viral plaque assays were performed on BAL samples obtained at 9 dpi as previously described (12). Briefly, Madin-Darby canine kidney cells were cultured on 6-well plates until reaching 70% confluence. Then, BALF samples were thawed, diluted 500× in PBS, with 0.1% BSA. A total of 200μL of the diluted sample was added to wells and incubated at 37°C for 1 hour. Then, cells were washed twice with PBS and covered with 2 mL of overlay consisting of a 1:1 mixture of 2% agarose and 2× DMEM with 2 μg/mL acetylated trypsin. Cells were incubated at 37°C for 72 hours to allow viral plaques to develop; overlay gels were then removed, and cells were fixed for 20 minutes in 70% ethanol and then stained for 20 minutes with 0.3% crystal violet solution. Viral plaques were then counted, and PFUs were calculated by the formula PFU = no. of plaques × dilution factor.

### RAW cell culture with ONP-302 exposure.

In total, 6 × 10^5^ RAW264.7 macrophages (ATCC, TIB-71) were plated in a 12-well cell culture plate and cultured overnight in RPMI-1640 (Invitrogen, 21870-100) media supplemented with FSC (Gemini, 100-106), and L-glutamine (Invitrogen, 25030-164). The next day, cells were treated with ONP-302 (2.5 μg/mL) or media alone (untreated) for 24 hours. Cell culture supernatants were then collected, and the concentrations of indicated cytokines and chemokines were assayed by Luminex (Mouse cytokine/chemokine magnetic bead panel, MilliporeSigma, MCYTOMAG-70K).

### Statistics.

All data are presented as mean ± SEM, except for Table 1, in which mean ± SD is shown. Statistical significance was determined using Student’s 2 tailed *t* test in GraphPad Prism. In data that were analyzed by multiple 2-tailed *t* tests (Figure 4), the comparison was made by 2-tailed *t* test with Bonferroni correction. In data that included with multiple comparison groups (Figure 7D), data were evaluated by 1-way ANOVA with Tukey’s multiple comparisons. *P* values are denoted in the figures or figure legends. *P* < 0.05 was considered significant. All graphs represent samples pooled from at least 2 independent experiments.

### Study approval.

Animal studies were conducted following the *Guide for the Care and Use of Laboratory Animals* (National Academies Press, 2011) and approved by the IACUC of the University of Michigan.

## Author contributions

WJK contributed conceptualization, data collection and analysis, access to data, and methodology, and he wrote the original draft. KMW performed flow cytometric studies and ELISA studies, performed data collection and analysis, and edited the manuscript. JC contributed conceptualization, data collection, and methodology, and she edited manuscript. TM contributed conceptualization and project administration. QX contributed methodology and particle preparation. MTB contributed conceptualization, funding acquisition, and project administration. JRP contributed methodology. AE contributed conceptualization, supervision, and data analysis, and he edited manuscript. DRG contributed conceptualization, supervision, funding acquisition, access to data, and data analysis, and he wrote and edited manuscript.

## Supplementary Material

Supplemental data

## Figures and Tables

**Table 1:**
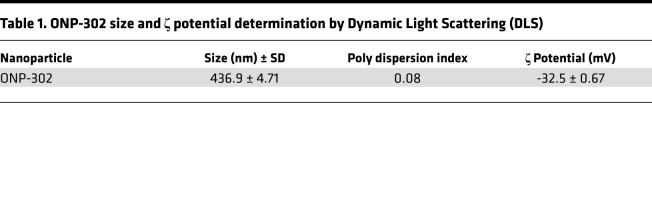
ONP-302 size and ζ potential determination by Dynamic Light Scattering (DLS)

## Version 1 06/23/2022

In-Press Preview

## Version 2 08/08/2022

Electronic publication
