# Supplementary material for: Nanoparticles reduce monocytes within the lungs to improve outcomes after influenza virus infection in aged mice
Source: JCI Insight. 2022 Aug 8;7(15):e156320. doi: 10.1172/jci.insight.156320 (PMC9462478; doi:10.1172/jci.insight.156320)
Supplement: Supplemental data [file jciinsight-7-156320-s147.pdf]

Supplemental Figures
Nanoparticles reduce monocytes within the lungs to improve outcomes after influenza virus infection in aged mice
Kelly et al

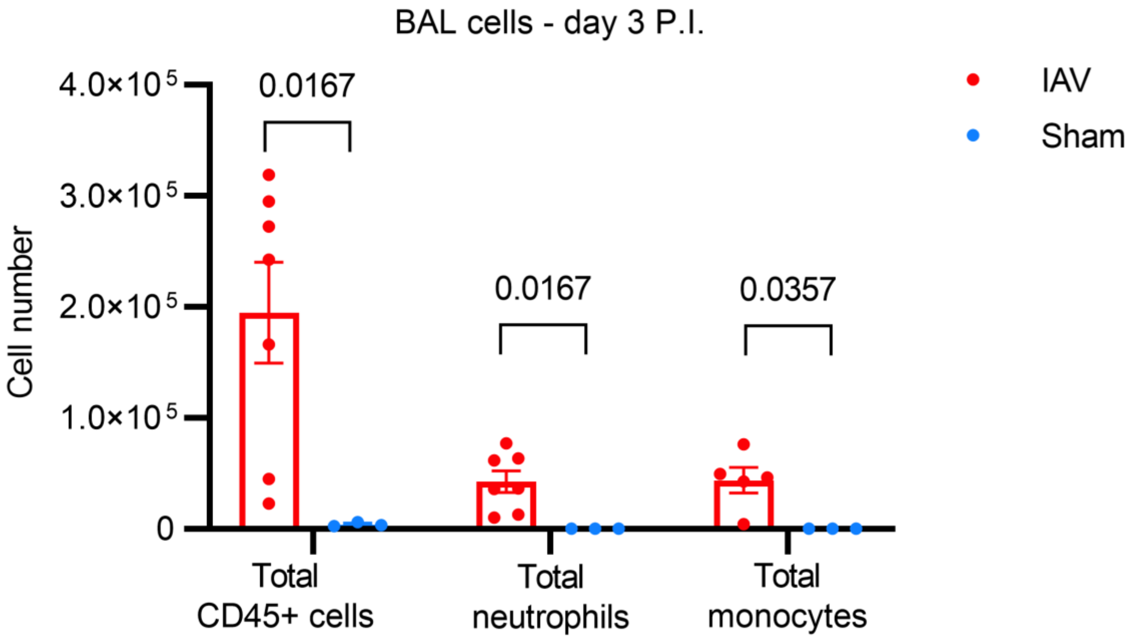

Supplemental Figure 1: Aged female (18-20 months of age) C57BL/6J mice were infected with 600 PFU IAV or sham infected. Bronchoalveolar Lavage (BAL) at 3 D.P.I. in sham and influenza-infected aged mice shows that influenza infected aged mice exhibited a significant increase in the absolute numbers of $CD45^+$ cells, neutrophils and monocytes compared to sham infected mice. Each data point represents a separate biological replicate. Statistical significance was determined using Student's t test. Significant p values are shown.

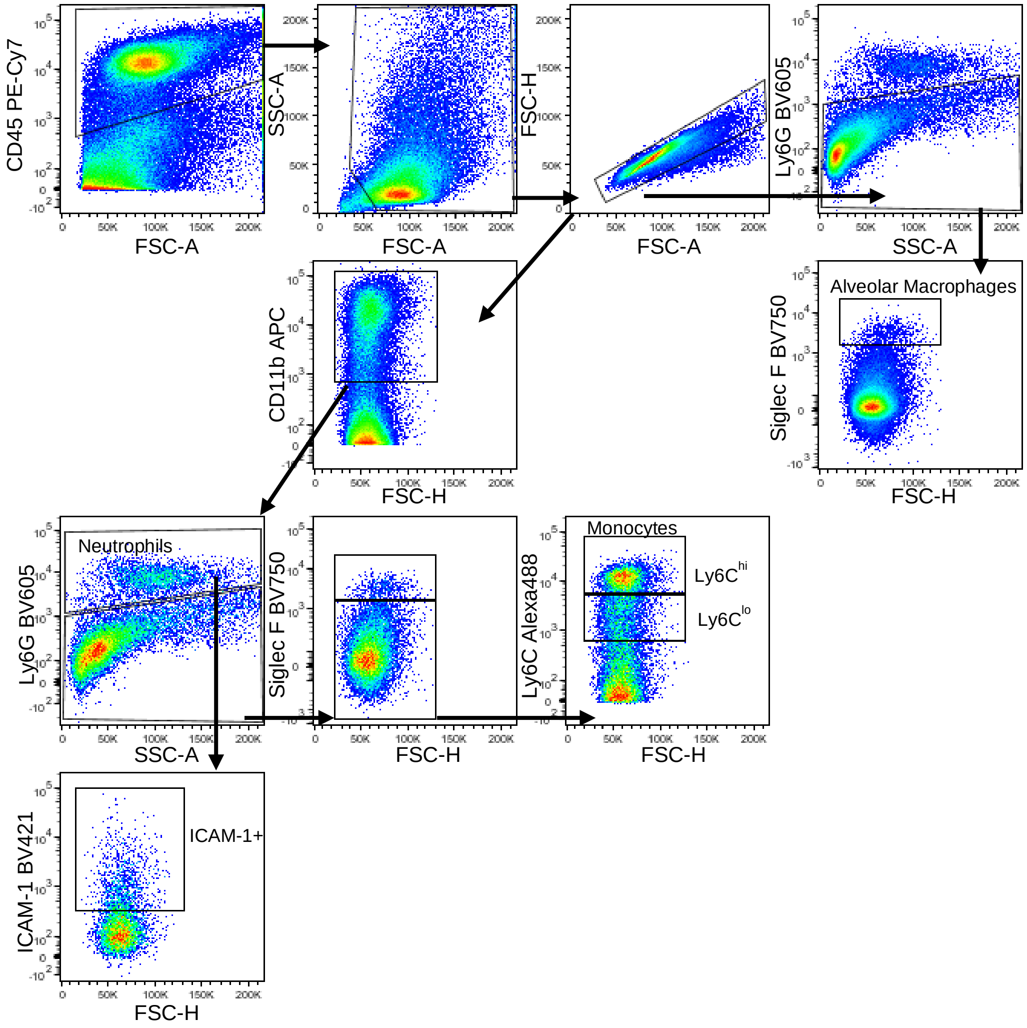

Supplemental Figure 2: Representative flow cytometric gating scheme for the identification of neutrophils, monocytes, alveolar macrophages and ICAM+ve neutrophils in mouse BAL, lungs, liver, and spleen.

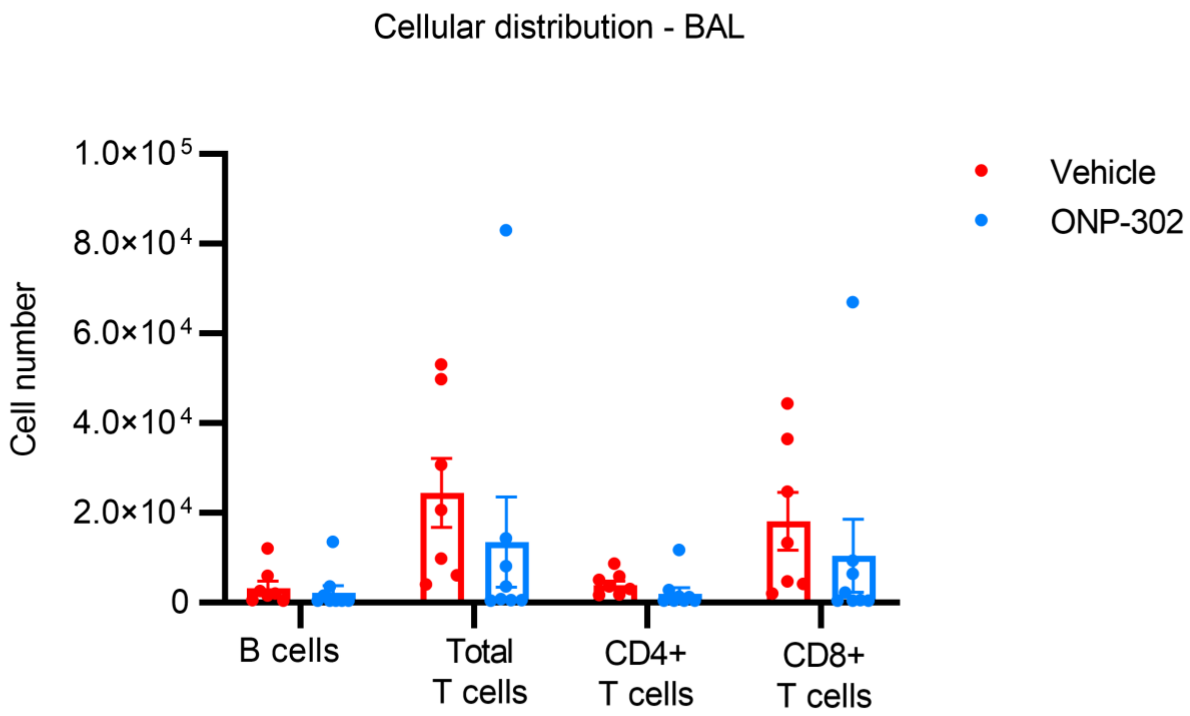

Supplemental Figure 3: ONP-302 do not impact the number of B and T cells within the BAL at 6 D.P.I. Aged female C57BL/6J mice were infected with 600 PFU IAV then administered ONP-302 daily, or vehicle control, via i.v., tail vein injection from day 3 D.P.I to either day 5 D.P.I. BAL was harvested at 6 DPI. The absolute number of B and T cells were determined via flow cytometry. Each data point represents a separate biological replicate.

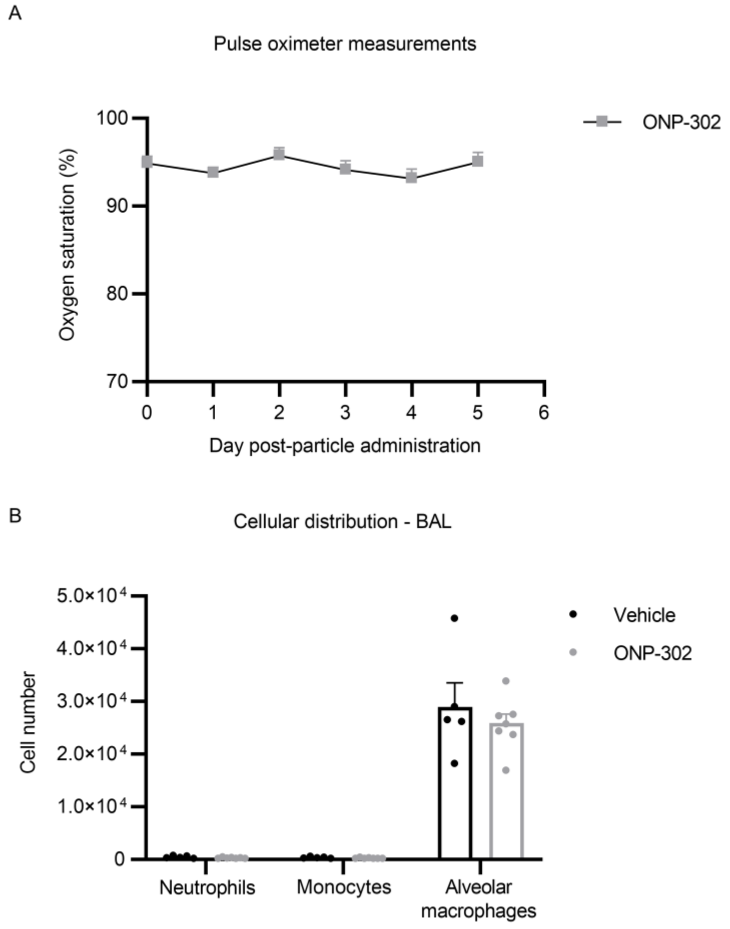

Supplemental Figure 4: ONP-302 do not impact oxygen saturation levels or induce influx of myeloid cells within the BAL. **A:** Oxygen saturation levels in ONP-302-treated and non-infected aged mice as measured by pulse oximeter (MouseStat Jr.). **B:** ONP-302 particles were administered for daily for 5 days via i.v., tail vein injection into non-infected mice aged mice. Two days after the last ONP-302 infusion, lungs were harvested, and BAL stained for neutrophils and monocytes via flow cytometric staining. N = 8 mice/group

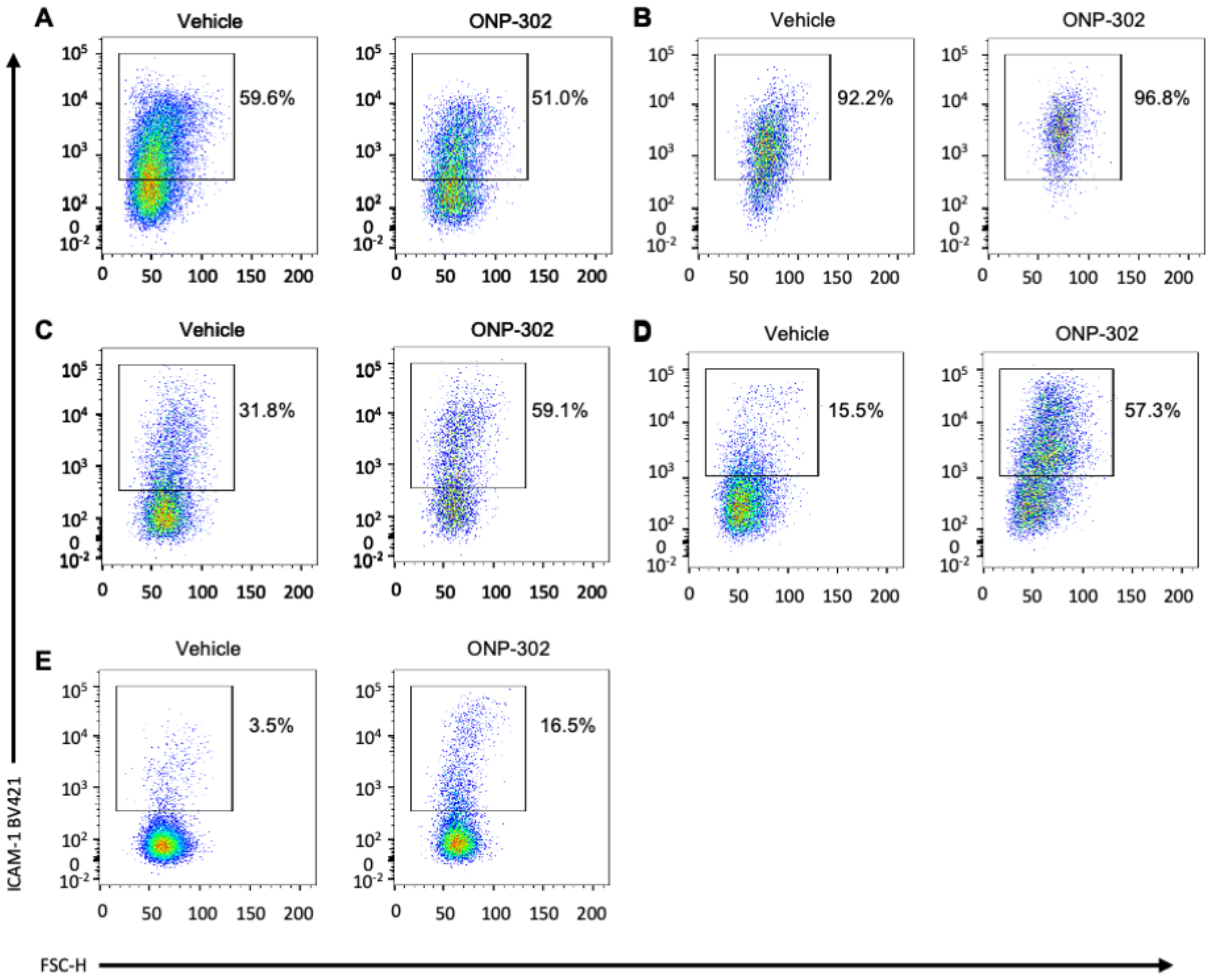

Supplemental Figure 5: Representative flow cytometric gating scheme for enumerating the frequency of ICAM-1+ neutrophils in different tissues.
